# Patients’ response during the co-circulation of multiple respiratory diseases in China—based on the self-regulation common-sense model

**DOI:** 10.3389/fpubh.2024.1365848

**Published:** 2024-02-29

**Authors:** Shanwen Sun, Yali Wang, Hailong Hou, Yuqi Niu, Yefan Shao, Linlin Chen, Xiaochun Zhang

**Affiliations:** ^1^The First Affiliated Hospital of China Medical University, Shenyang, China; ^2^School of Nursing, China Medical University, Shenyang, China

**Keywords:** respiratory infections, public health events, self-regulation common sense model, KJ method, qualitative research

## Abstract

**Background:**

Following the COVID-19 pandemic, another large-scale respiratory epidemic has emerged in China, causing significant social impact and disruption. The article is to explore the patients’ psychological and behavioral responses to the enhancement of healthcare quality.

**Methods:**

Based on the five dimensions of the Self-Regulation Common-Sense Model, we developed an interview outline to explore the process by which patients identify disease symptoms to guide action plans and coping strategies. The researchers used a semi-structured interview format to simultaneously collect data online and offline. This study gathered data from 12 patients with mixed respiratory infections, comprising 58% females and 42% males; the average age was 30.67 years (SD 20.00), with 91.7% infected with two pathogens and 8.3% with three. The data analysis employed the KJ method, themes were inductively analyzed and categorized from semi-structured interview results, which were then organized into a coherent visual and logical pathway.

**Key results:**

The study identified 5 themes: (1) Autonomous Actions Prior to Seeking Medical Care; (2) Decision-Making in Seeking Hospital Care; (3) Disease Shock; (4) Public Crisis Response; (5) Information Cocoon.

**Conclusion:**

The pandemic of respiratory infectious diseases has not ceased in recent years. Following the COVID-19 pandemic, China is now facing a trend of concurrent epidemics involving multiple respiratory pathogens. This study centers on patients’ health behaviors, exploring the potential relationships among various factors that affect these behaviors. The aim is to provide references and grounds for the improvement of healthcare services when such public health events reoccur.

## 1 Background

Acute Respiratory Infections (ARI) are contagious diseases caused by respiratory pathogens such as viruses, bacteria, and mycoplasma (1). With the onset of autumn and winter, respiratory pathogens exhibit a concurrent epidemic trend, causing considerable fluctuations and impacts in society (2). Due to various potential mechanisms of interaction among pathogens, there is a possibility of superimposed infections, leading to more complex and severe clinical symptoms (3). Numerous studies have reported cases where patients were simultaneously infected with multiple respiratory pathogens (4–6). These include Respiratory Syncytial Virus (RSV), Influenza A Virus (IAV), Influenza B Virus (IBV), Coronaviruses (CoV), Adenovirus (AdV), and Mycoplasma, among others. Large-scale respiratory infections significantly escalate the demand for medical resources, potentially leading to an overload of the public health system (7).

The recent COVID-19 pandemic has been one of the most severe public health crises in recent years. Unlike other disaster events, COVID-19, as a newly emerged infectious disease at that time, is characterized by strong pathogenicity, high infection rate, rapid transmission, and a wide range of impact, leaving an indelible impact on society (8, 9). This phenomenon significantly influenced public psychology and behavior. Studies have demonstrated a high prevalence of Post-Traumatic Stress Disorder (PTSD) among COVID-19 patients, with frequent occurrences of negative emotions such as panic, anxiety, and depression (10). Additionally, isolation, business suspension, and school closures negatively impacted the mental well-being of professionals and students (11, 12). Conversely, this experience enhanced public health literacy, fostering improved psychological and behavioral responses during health crises (13). When facing the resurgence of similar respiratory diseases, the psychological and behavioral changes in individuals become a matter of profound consideration. Researchers investigated the psychological responses of patients to influenza and common colds post-COVID-19 pandemic (14). Findings indicate that patients now face illnesses more positively and seriously. However, there is a significant portion of the population experiencing panic and concern due to the difficulty in distinguishing between the clinical symptoms of these diseases and COVID-19. Currently, few studies focus on patients’ psychological and behavioral responses to similar public health events, which is the primary focus and research interest of this study.

Previous quantitative studies exploring public psychology and behavior during public health events often lacked a deep understanding of complex social phenomena. This is because the hypotheses in quantitative research often stem from the researcher’s preconceived perspectives, exploring the interrelationships, influence pathways, and moderating effects between variables. For instance, researchers have attempted to analyze the relationship between patients’ medication adherence, health literacy, and self-care behaviors in the post-pandemic era, based on hypotheses formed from previous studies (13). However, exploring unfamiliar or vague psychological phenomena is challenging in this approach. This is where qualitative research stands out, offering insights into unexplored areas. In fields where qualitative research is scarce, employing qualitative analysis to investigate more potential relationships is essential. Therefore, this study adopts a qualitative approach, aiming to delve into the rich and complex psychological and behavioral changes involved. The common-sense model of self-regulation (CSM) is a theoretical model where patients initiate and maintain self-regulation based on common sense (15). They determine whether to adjust or modify their coping strategies and illness representations by evaluating the outcomes, forming a dynamic and continuous feedback system. Hence, the model will be incorporated in the development of the interview outline.

In addition to other qualitative research methods, phenomenological studies often employ Colaizzi’s 7-step analysis method for data analysis, a process that can be tedious and complex in categorizing and extracting themes. The KJ Method, developed by Dr. Kawakita Jirou during his organization of Nepalese expedition data, also known as the affinity diagram method, offers an alternative (16). This method involves synthesizing complex and initially unstructured ideas or facts based on their mutual affinity, corresponding to the problem awareness and rooted in the unity of the internal object. It facilitates intuitive data organization, clarifying relationships and information pathways. This method has become one of the new seven quality management tools in China. Therefore, this study employs this method for analysis.

## 2 Methods

### 2.1 Study participants

This study employed purposive sampling to collect data. From September to December 2023, patients with mixed respiratory infections were recruited offline at the outpatient department of the First Affiliated Hospital of China Medical University. Online recruitment was conducted with compensation. Sample selection followed the principle of maximum variation, and the sample size was determined based on data saturation. This study has received ethical approval from the ethics committee of the first affiliated hospital of China Medical University. Approval No. [2020]394.

Inclusion Criteria: (1) Patients diagnosed with infections by two or more respiratory pathogens, confirmed through respiratory virus antigen testing, bacterial cultures of respiratory specimens, and PCR-DNA testing for *Mycoplasma pneumoniae* and *Chlamydophila pneumoniae* (17). (2) Patients who have given informed consent and signed the consent form.

Exclusion Criteria: (1) Patients with complications in other systems and critical conditions. (2) Patients with language communication barriers.

#### 2.1.1 Development of the interview outline

The research team consisted of 10 members, including one senior expert in the field of respiratory and critical care, six master’s degree students, one respiratory outpatient medical staff member, and two experts who are familiar with the KJ Method. Based on the research objective and a review of relevant domestic and international literature, the team developed a preliminary interview outline grounded in the five illness representation dimensions of the common-sense model of self-regulation. An initial trial interview with two patients was conducted, and the results were used to refine the outline. After consultation with experts, the final interview outline was established, which mainly included the following contents (Table 1).

**Table 1:** Interview outline.

Interview outline	Supplementary questions
1. How did you realize that you were ill? (Identity Dimension)	Describe your initial symptoms. How were they different from common cold symptoms? What methods did you use to make an initial assessment?
2. Can you briefly describe the timeline of your illness? (Timeline Dimension)	Detail the timeline of your illness: When did the symptoms start, how long did you self-manage before seeking medical help, when did you seek medical attention, and what do you estimate as the recovery time? If applicable, explain any reasons for delayed medical consultation
3. What do you think caused your illness? (Cause Dimension)	What specific factors do you believe caused your illness? Consider aspects such as physical condition, emotional state, seasonal climate, or the evolution of infectious diseases
4. What measures have you taken to control and alleviate your symptoms? (Control Dimension)	Are you aware of specific treatments, dietary adjustments, lifestyle changes, and preventive measures? If not, specify what kind of assistance or information you need
5. What impact has the illness had on you? (Consequence Dimension)	Explain the specific impacts of the illness, both physical and psychological
6. What are your thoughts on the current trend of multiple respiratory pathogens co-circulating?	Discuss your perspective on the concurrent prevalence of multiple respiratory pathogens at the individual, familial, and societal levels

#### 2.1.2 Data collection method

Offline interviews were conducted in a tranquil room, all by the same interviewer using semi-structured, in-depth methods. For interviewing special groups like children and older adult, interviewers should use simple, understandable language and ensure questions are age and cognition-appropriate for efficiency and brevity. Additionally, allowing family members of children and older adult to supplement responses from the interviewee’s perspective ensures reliability and comprehensiveness of the data. Ethical guidelines, including obtaining consent from parents or guardians, must be followed when interviewing children. Interviewers should confirm unclear responses or questions promptly through restatement. Online interviews, through one-on-one voice calls, followed similar procedures.

Preparation involved reviewing topic-related materials for focused, quality interviews. Participants were informed about the study’s aim, content, and voluntary nature, ensuring confidentiality before signing consent forms. With consent, the entire process was recorded, capturing both audio and key verbal/non-verbal cues. The duration of each interview was controlled to be within 20–30 min.

#### 2.1.3 Quality control

The expert team members, seasoned in respiratory and critical care, are proficient in the KJ Method. The group’s graduate students, well-versed in qualitative research from their master’s programs, excel in communication and data organization. This study ensures the representativeness of its subjects. In the selection process, subjects are chosen based on criteria such as typicality, diversity, or homogeneity to enhance the authenticity of the data. During the research, triangulation methods are employed, including data triangulation (collecting data at different time points, from different locations, and from subjects with different characteristics), investigator triangulation (where multiple researchers analyze the same data, e.g., KJ method), methodological triangulation (combining various data collection methods such as interviews and observations), and analysis triangulation (involving multiple analysts in repeated analysis, induction, brainstorming, and constantly comparing findings with original data using methods like KJ method). These approaches aim to improve the validity and the rational and logical interpretation of the data, thereby enhancing its credibility. Through self-questioning and reflection, researchers strive to maintain an unbiased stance, exploring and understanding the experiences, processes, or cultures studied from the perspective of the subjects. Post-data collection, responses were verified with interviewees, securing data authenticity and precision.

#### 2.1.4 Data analysis method

Within 24 h after each interview, the recorded content was transcribed into text. The specific methods were as follows: (1) Two researchers independently and thoroughly analyzed all relevant materials, listening to the recordings multiple times to ensure a deep understanding of the interviewees’ statements (2). Key statements from the interviewees were selected. These statements were then coded into concise phrases or short sentences, aimed at extracting crucial information. The original descriptions by the interviewees were noted in parentheses after coding to retain the context (3). Card preparation: The coded content was recorded on homemade small cards, each measuring approximately 3 cm × 4 cm (4). Card editing and theme extraction: In a quiet room, using a large table, all small cards were spread out. The team members read the content of each card carefully, at least three to four times, to deeply understand the meaning of each code. Cards with redundant meanings were discarded through brainstorming. Cards with similar contents were grouped together, forming several clusters. New cards were made for each cluster, summarizing the theme and noting it on colored cards placed in the respective group. Similar cluster themes were further categorized into medium themes, marked with different colored paper. Finally, related medium themes were integrated into major themes (5). Creating a KJ diagram: The categorized cards were examined from a macro perspective to arrange them in a visually and logically coherent layout. A KJ diagram was first hand-drawn, then refined using computer tools. The themes at various levels and the interviewees’ descriptions regarding these themes were organized and returned to the interviewees for verification.

## 3 Results

### 3.1 General information

The study reached thematic saturation after 12 interviews. Demographic data are as follows: 58% of the participants were female, and 42% were male; the average age was 30.67 (SD 20.00); 75% lived in urban areas, while 25% resided in rural areas; 91.7% of the participants were infected with two pathogens, and 8.3% were infected with three pathogens (Table 2).

**Table 2:** Demographic data.

No.	Gender	Age	Region	Residence	Occupation	Infection type
1	Female	26	Jiangsu	Urban	Editor	IAV + Mycoplasma
2	Male	14	Liaoning	Urban	Student	Mycoplasma + Bacteria
3	Female	22	Hunan	Urban	Nurse	IAV + Bacteria
4	Female	27	Beijing	Urban	Teacher	IAV + RSV
5	Male	25	Liaoning	Urban	Teacher	IAV + Mycoplasma + RSV
6	Male	32	Shanghai	Urban	Manager	COVID-19 + Mycoplasma
7	Female	8	Hubei	Urban	Student	IAV + Mycoplasma
8	Female	62	Liaoning	Rural	Retired	COVID-19 + IAV
9	Male	33	Liaoning	Urban	Clerk	IAV + Mycoplasma
10	Male	81	Liaoning	Rural	Farmer	IAV + Bacteria
11	Female	25	Beijing	Urban	Editor	IAV + RSV
12	Female	13	Jiangsu	Rural	Student	Mycoplasma + Bacteria

### 3.2 Comprehensive results

**Figure 1:**
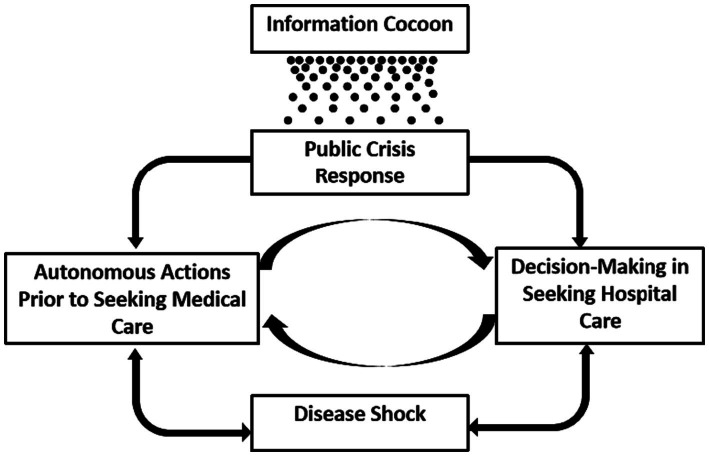
Pathways of influence between themes.

The study initially included 266 entries of cards. After excluding semantically redundant entries, 72 cards remained. Following a brainstorming process, the final categorization used 34 small group title cards and 13 medium group title cards, ultimately resulting in 5 major group title cards, representing 5 themes. The specific details are as follows (Figure 1).

### 3.3 Theme one: autonomous actions prior to seeking medical care

#### 3.3.1 Individual physical perception

Individuals’ perception of changes in their physical condition aids in the timely identification of potential health issues. For instance, ID5 stated, “I believe prompt bodily awareness is crucial; I know best about my reactions,” and ID3 stated, “As a nursing intern who works in a hospital, I am quite sensitive to changes in my body. I notice something is wrong as soon as symptoms appear.”

However, there are cases where individuals have a weaker perception of their illness, leading to confusion between discomfort and other physical symptoms, resulting in misjudgment of symptoms and delayed treatment. For example, ID3 stated, “I just felt a little discomfort in my throat, maybe due to fatigue from a business trip, and did not pay much attention.” ID4 shared, “I had headaches, back pain, and stomachaches. Initially, I thought these might be symptoms of menstruation and did not take them seriously.”

#### 3.3.2 Preliminary self-diagnosis

Upon experiencing symptoms, people often make preliminary diagnoses by consulting various sources. For instance, ID11 mentioned, “I checked with a friend who had contracted influenza around the same time, asking about his symptoms for confirmation.” ID1 said, “I felt that the symptoms of this illness were similar to when I had COVID-19, which made me more alert.” ID6 stated, “I do not know much about this illness; I could only look up scientific information online to judge if I had it.”

#### 3.3.3 Speculation on the cause of illness

Individuals’ subjective judgments about their health include speculations about the causes of their illness. For example, ID4 said, “I often stay up late, which might have weakened my immune system.” ID6 expressed, “The main reason could be a lack of proper protection, especially if people around me showed similar symptoms and I wasn’t wearing a mask at that time.”

#### 3.3.4 Self-care prior to hospital visits

In the early stages of the illness, after making preliminary judgments, people tend to employ self-management strategies for experiential treatment and coping. For example, ID1 said, “One night I had a fever and took two antipyretic pills based on my experience.” ID3 mentioned, “As soon as I noticed symptoms, I bought antigen self-test kits.” ID5 stated, “Sometimes we just consult doctors online, which usually resolves general issues.”

### 3.4 Theme two: decision-making in seeking hospital care

#### 3.4.1 Motivations and barriers to hospital medical care

Patients with mixed infections often experience persistent symptoms. When symptoms become unmanageable, it triggers the decision to seek medical care. For example, ID2 stated, “He later developed a fever of over 39 degrees and felt much worse, so we hurried to the hospital for a check-up.” ID1 mentioned, “The next day I could not bear it anymore and rushed to the hospital in the afternoon for an IV.” Persuasion from others can strengthen this decision. ID1 said, “I initially decided to endure it and focus on work, but my boss advised me to go to the hospital if the symptoms got worse, which made me waver.”

However, social pressure creates a reluctance to seek medical care, leading to apprehensions. ID9 expressed, “Taking sick leave definitely affects work; I was afraid of causing trouble with my boss.” ID2 noted, “To some extent, if others choose to attend school while sick, we are put in a difficult position, creating a vicious cycle.”

#### 3.4.2 Hospital visit experiences and perceptions

The development and advancement of modern medical resources have brought convenience and enriched the healthcare experience. For instance, ID5 said, “There’s a medical chaperone service in hospitals now, which I think is a great trend.” ID5 also mentioned, “One good thing about mobile appointments is that you do not have to queue for registration, as it’s already done on the phone.”

However, various difficulties in the medical consultation process lead to a less favorable experience for patients. Objective factors pose significant challenges, such as ID5 stating, “Hospitals are too expensive, and outpatient services are not reimbursed by medical insurance.” ID8 noted, “And you know, getting a registration in big hospitals like yours is inconvenient; it’s always crowded. I’d avoid coming if possible.” ID11 added, “Some older adult may not want to come to the hospital because of its complicated procedures.”

On the other hand, patients also focus on their subjective feelings during the consultation process. For example, ID5 mentioned, “Sometimes nurses, receptionists, and doctors might be too tired and in a bad mood, which deters patients from wanting to seek treatment.”

### 3.5 Theme three: disease shock

#### 3.5.1 Psychological reactions during illness

The experience of mixed infections can have a negative impact on patients’ psychology, not only manifesting in negative responses to their own physical conditions but also leading to concerns and worries about family and friends. For example, ID4 shared, “When I was sick, I felt impatient and irritable.” ID11 expressed, “Especially regarding our family members and close ones, there’s definitely a lot of worry about the influenza virus.”

However, some patients managed to maintain an optimistic attitude, as ID2 stated, “Even if really infected, it’s important to stay relaxed.” Additionally, some patients highlighted that family support could bring positive inner strength. For instance, ID10 said, “My children are really good to me, and my family supports me. This makes me feel warm inside.”

#### 3.5.2 Impact of illness on behavior

The experience of mixed infections also affects individuals’ behaviors, not only in terms of lifestyle changes but also in terms of future vigilance and preventative measures. For instance, ID mentioned, “After falling ill, I try to sleep early to ensure enough rest.” ID2 said, “Since getting sick, we think every day about how to eat healthier.” ID12 noted, “I advised him to drink more hot water after starting medication.” ID1 added, “So, I pay special attention to the intake of Vitamin C, believing it to be very important.”

This is also reflected in future warning and preventive behaviors. For example, ID4 stated, “Since showing symptoms, I’ve been continuously wearing a mask.” ID7 mentioned, “We will be more cautious about keeping warm to avoid getting cold in the future.” ID5 shared, “I stock up on some medicines at home, just as a precaution, even if they are not used immediately.”

### 3.6 Theme four: information cocoon

#### 3.6.1 The “information cocoon” has impacted public perception

Harvard University Professor Keith Sunstein introduced the concept of the “information cocoon” in 2006, suggesting that the public, focusing only on information of interest, will ultimately confine themselves within a cocoon—much like a silkworm (18). The societal issues during the epidemic are mainly present in the domain of online information. This encompasses not just the gap in information but also doubts about the authenticity of information. For example, ID1 observed, “I find the news and trending topics online to be quite insensitive. The outbreak has been serious since October or August, but it only started getting attention in late November, which feels like a delay in information.” ID6 commented, “Prevention information is not as widespread as it was during the initial COVID-19 outbreak.” ID1 further stated, “In the early stages, the lack of official communication led to the spread of rumors, including many speculations about the disease.” ID3 added, “The reliability of information from online channels varies; some may be correct, but there is also misleading information.”

### 3.7 Theme five: public crisis response

#### 3.7.1 Public concerns and panic toward the epidemic

Faced with recent epidemic outbreaks, the public has developed fears and confusion about the future. ID9 said, “We are worried that if we have to face such diseases every year, it feels quite pessimistic.” ID11 expressed, “I’m really scared about the recent reports of flu mutation,” and “We are unsure when the virus will be completely eradicated.”

The reality also includes the shadow and trauma left by the COVID-19 pandemic. For instance, ID5 shared, “I’m concerned that we might have to go back to wearing masks every day at work and school like during COVID-19.” ID3 noted, “During COVID-19, people hoarded related medicines, causing price hikes and shortages, making it difficult for those who really needed them to get access. I’m worried about this happening again.”

#### 3.7.2 Public confidence and affirmation in society and the state

In response to recent epidemics, the public has also shown positive and optimistic attitudes toward the government and the nation. ID11 said, “I believe our country now has more mature experience and a more complete system in place.” ID9 stated, “I think our medical resources are sufficient now, and masks and medicines are available.” ID10 expressed, “I always feel that no matter how big the difficulty, as long as we are united, we can overcome any obstacle.” ID9 added, “As for work and school suspensions, if necessary, we will cooperate. There’s no particular resistance to it.”

#### 3.7.3 Shift in health awareness

Following the global sweep of the COVID-19 pandemic, the public’s perception of respiratory epidemics has changed to varying degrees. ID11 noted, “After experiencing last year’s pandemic, I think people are not as panicked as before and can view things more rationally now.” ID11 also mentioned, “Having gone through the COVID-19 pandemic, there’s been a significant increase in health awareness among everyone.” ID9 stated, “After the pandemic, we feel that we are more inclined to enjoy life and not worry too much about other things.”

#### 3.7.4 Expectations for society

The public hopes for improvements in health facilities. For example, ID5 suggested, “Every hospital should have a flowchart of the consultation process at the entrance, so people can take a photo and understand it better, to avoid confusion.” ID5 also said, “There should be some convenient testing centers in communities, like during the COVID-19 pandemic.”

There is also a desire for the timely dissemination of simple and understandable medical information. ID3 expressed, “Major platforms need to publish simple guidance, easy to understand, to help the public judge symptoms and handle them straightforwardly.”

The call for vaccination, especially among vulnerable groups, has become a societal response. ID3 stated, “There are influenza vaccines available now, but we still need to encourage more people, especially older adult and children, who are more susceptible, to get vaccinated.”

### 3.8 Analysis of the interactions among the themes

Firstly, the health threat posed by the disease directly influences patients’ decisions regarding medical actions. The choice of medical behavior, in turn, directly drives the recovery or exacerbation of the disease, highlighting their interdependent relationship.

During the initial phase of illness, patients tend to first assess and manage their condition independently, deciding on further medical action based on the feedback from this self-treatment. On the other hand, the perceived benefits and barriers encountered during medical visits also influence future healthcare decisions. These two aspects continually interact throughout an individual’s life, forming a cyclical relationship.

The COVID-19 pandemic has had a profound impact on the public’s physical and mental health, altering their crisis response to similar public health events. The prevalence of online information as a key source and the emergence of information cocoons significantly influence this response. The phenomenon of ‘information cocoons’ has a permeating influence on the public’s response to communal crises. In this context, patients may gain a deeper understanding of health and illness, affecting their self-diagnosis capabilities and medical behavior, creating a causal relationship between public crisis response and medical behavior.

## 4 Discussion

Our study results align with the Self-Regulation Common-Sense Model’s theoretical framework. The experience of the COVID-19 pandemic triggered psychological and behavioral responses in individuals when faced with similar situations, leading to the adoption of health behaviors. A key focus of our research is the decision-making process between health self-management and seeking medical help. This relationship is influenced by a combination of factors. We also found that in today’s digital age, the internet greatly facilitates the medical process. However, the formation of information cocoons has become a significant factor affecting various aspects of a patient’s illness experience, a topic seldom mentioned in other studies.

In this study, we have conducted a more in-depth exploration of these crucial aspects, aiming to provide insights for the improvement of future healthcare systems.

### 4.1 Impact of experiencing public health events on individuals

Individuals who experience public health events may undergo significant changes on multiple psychological and behavioral levels. Zhang et al. (19) suggests that in the first personal experience of a sudden health event, an individual’s risk knowledge system is underdeveloped, unable to initiate protective measures, but there is a mutual construction of knowledge and perception. In the next similar experience, risk protection measures are more likely to be activated. Sayeed et al. (20) argues that the experience of contracting COVID-19 leads to a shift in participants’ values, where individuals may have already associated specific adverse experiences with negative emotions, a connection that is psychologically reinforced and persists. Additionally, this fear and anxiety might be subconsciously “learned” and triggered when faced with similar situations. In this study, people expressed worry, fear, and confusion regarding the recurring epidemics of respiratory infectious diseases (21). Conversely, some views also expressed optimistic and positive attitudes (22). After experiencing public health events, “psychological resilience” is enhanced, manifesting as increased “social cohesion” from the positive adaptation and recovery ability cultivated in adversity, and a strengthened community consciousness and trust in social institutions after facing common challenges (23, 24).

The experiences following public health emergencies are closely related to appropriate protective measures (19). Therefore, to maximize the role of experience, emphasizing health education post-public health emergencies is crucial. This approach ensures that individuals are better prepared and informed, potentially leading to more effective responses in similar future situations.

### 4.2 Comprehensive decision-making in medical behavior

Bodily perception plays a crucial role in the identification and management of illness, yet it is also subject to the complex influences of biological, psychological, and social environmental factors (25). These factors intertwine to shape individuals’ perception and interpretation of disease symptoms. However, common respiratory diseases often have similar initial symptoms, and some non-specific symptoms like fatigue, mild cough, and headache can be common features of various illnesses, making it more challenging for the general population to identify and differentiate specific diseases (3).

The Common-Sense Model of Self-Regulation provides a theoretical framework for explaining how individuals interpret and respond to health threats. They assess symptoms based on their cognitive models, attempting to determine the identity, cause, and potential consequences of the symptoms, and decide their response accordingly (26). If they believe the symptoms can be managed through self-regulation, they might not seek professional medical help. Only when the disease does not improve or becomes more severe does it draw patients’ attention. van Eeuwijk et al. (27) found that most older adult people seek medical help for rheumatic diseases as it impedes their activities. However, when the disease’s impact on their daily life is minor, it does not trigger the action to seek medical care. Differing from previous studies, this research found that the pressures of work and study also contribute to delayed medical consultation. Interviewees believed that seeking medical care for illness would affect their study or work progress, a concern stemming from the consideration of potential time and energy loss due to medical visits, which is likely closely related to the fast-paced contemporary societal environment (28).

Medical behavior, referring to the actions individuals take to seek professional medical services when facing health problems or diseases (29), has evolved with modern medical advancements, enhancing accessibility, efficiency, and patient experience. Through electronic health records, online appointment systems, telemedicine, and mobile health apps (mHealth apps), patients can more conveniently manage their health information, schedule appointments, and receive remote consultations (30). Statistics show that the scale of online medical service users has reached 276 million in China, accounting for 29.4% of internet users (31). Recently, Online Health Communities (OHCs) have emerged, offering platforms that connect doctors and patients for consultations on health issues and disease treatments anytime and anywhere (30). Current challenges such as medical staff shortages and high patient volumes impede the development of high-quality outpatient services. The rise of companion care services not only improves patients’ medical experiences but also promotes the fairness of medical services, ensuring everyone receives appropriate medical care (32).

The experience and feelings during the medical process are also important topics for exploration. The complexity of the medical process, such as the difficulty of registration, economic factors, and the attitude of medical staff, is considered a major challenge in hospital visits. This is similar to Yao’s research (33), where lack of medical resources, poor service attitudes, and complex procedures are among the top five reasons for complaints. Many people are reluctant to visit public health institutions due to complicated administrative procedures, one-sided communication from health professionals, and long wait times (34). Iskandarsyah supports that treatment cost is one of the barriers to treatment (35). Only by fully understanding the current status and characteristics of medical behavior can targeted reforms and improvements be made in the medical and health sector and the medical insurance system, better meeting people’s medical needs. Due to the lack of medical knowledge among non-medical personnel, patients prefer to receive health guidance that is simpler and more intuitive, as this facilitates the acquisition and dissemination of knowledge. Additionally, vaccination emerged as a notable topic in the interview results, with respondents expressing their anticipation for the development of targeted vaccines, as well as the desire for widespread vaccination among susceptible populations such as children and older adult.

The 2018 National Health Statistics Survey showed that the overall satisfaction rate of urban and rural residents with outpatient services was 80.0%, a 3.5 percentage point increase from 2013, indicating improved patient experience (36). Satisfaction with medical costs, attitudes of medical staff, and the environment of medical institutions increased by 5.8, 5.2, and 3.3 percentage points, respectively, from 2013. In recent years, the state has issued relevant documents aimed at improving and perfecting the outpatient medical insurance system (37). This reflects the effectiveness of recent public health policies and resource investments, showing progress in medical technology, service process optimization, improved doctor-patient communication, and the successful implementation of digital and modern medical services. However, it also highlights areas that need continued focus and optimization. Additionally, in the context of an increasingly comprehensive medical insurance system and gradually abundant medical resources, a new research direction is provided to further improve patient visitation rates.

### 4.3 Breaking the information cocoon

Studies indicate that during sudden public health events, people increasingly rely on and are confined to online channels for information. Television programs, government websites, news media, and government WeChat public accounts are considered the primary sources for personal protection knowledge. Among these, government media, WeChat public accounts, and authoritative medical experts are deemed the most reliable sources by the public (38).

In the internet era, the geometric growth of information has led to an environment of information overload. The constant influx of vast amounts of information increases public cognitive load and decision-making difficulties, complicating the online environment. Homogenized information leads to a narrowing of information sources, resulting in groupthink and polarization (39). With the proliferation of social media platforms, initial signs of information narrowing can easily trigger a series of online controversies, rumors, and misinformation issues.

The 2011 revised “Emergency Regulations for Public Health Emergencies” Article 25 states, “The state establishes an information release system for emergencies, requiring that information release should be timely and accurate (40).” The public increasingly relies on government transparency. If the government lacks a robust information disclosure mechanism, the public is prone to fall into the cocoon of false information. Therefore, to reduce the impact of epidemics on information cocoons, society should mandate social media and online platforms to take responsibility for content moderation, establish strict review mechanisms, and promptly remove violations. Encouraging network platforms to disclose their content management standards and processes can enhance public trust in platform monitoring actions. Clear online content standards and laws should be formulated to ensure that publishers follow regulations, with strict penalties implemented for the illegal spread of false information.

### 4.4 Limitations

The limitations of this study primarily arise from the inherent subjectivity of qualitative research, significantly related to the researchers’ cognitive abilities, knowledge reserves, and capacity to analyze data. Moreover, the inclusion of children and older adult, who have limited understanding and expressive capabilities, poses a challenge. Even though researchers encouraged family members to accompany and supplement responses, whether the full extent of participants’ thoughts was truly uncovered remains a matter for consideration. Future research, building on this study, could continue to explore potential behaviors and motivations, conducting longitudinal studies to track and analyze changes in patient behavior over time, and developing and evaluating educational and health promotion strategies tailored to the needs of patients at different stages. Optimizing the medical process according to public needs and societal shortcomings can enhance the quality of medical services and the completeness of the medical system during public health events.

## 5 Conclusion

The peak period of the COVID-19 pandemic has passed, yet it has caused destructive impacts globally. The prevalence of respiratory infectious diseases has not ceased, and these diseases vary in impact due to regional, seasonal, and climatic factors, making complete prevention challenging. Recently, China experienced a significant outbreak of overlapping respiratory diseases, affecting many people. Future similar events, anticipated or not, may occur.

Understanding these responses is crucial for decision-makers to develop more effective public health emergency plans. Effective management of public health events and reducing negative psychological impacts are essential for maintaining social stability and promoting public health. At the onset of illness, patients often make comprehensive responses and decisions based on past experiences, existing knowledge, perception of symptoms, and external verification, revising and adjusting their coping strategies accordingly. This study categorizes patients’ medical behaviors into pre- and post-medical consultation phases, revealing potential health issues and risky behaviors, aiding in early identification and intervention. Additionally, it helps to more clearly understand patients’ behavioral patterns and motivations at different stages, as well as their varying needs and actions throughout the medical process, thus providing more personalized medical services.

## Data availability statement

The original contributions presented in the study are included in the article/supplementary material, further inquiries can be directed to the corresponding author.

## Ethics statement

The studies involving humans were approved by Ethics Committee for Medical Science Research, The First Hospital Affiliated with China Medical University. The studies were conducted in accordance with the local legislation and institutional requirements. Written informed consent for participation in this study was provided by the participants’ legal guardians/next of kin. Written informed consent was obtained from the individual(s), and minor(s)’ legal guardian/next of kin, for the publication of any potentially identifiable images or data included in this article.

## Author contributions

SS: Conceptualization, Data curation, Formal analysis, Investigation, Methodology, Project administration, Writing – original draft, Writing – review & editing. YW: Data curation, Formal analysis, Investigation, Methodology, Writing – original draft. HH: Data curation, Formal analysis, Methodology, Validation, Writing – original draft. YN: Data curation, Formal analysis, Methodology, Visualization, Writing – original draft. YS: Data curation, Formal analysis, Methodology, Writing – original draft. LC: Data curation, Formal analysis, Methodology, Writing – original draft. XZ: Resources, Supervision, Validation, Writing – review & editing, Writing – original draft.
